# Supplementary material for: Identifying Novel Osteoarthritis-Associated Genes in Human Cartilage Using a Systematic Meta-Analysis and a Multi-Source Integrated Network
Source: Int J Mol Sci. 2022 Apr 15;23(8):4395. doi: 10.3390/ijms23084395 (PMC9030814; doi:10.3390/ijms23084395)
Supplement: Supplementary file 1 [file ijms-23-04395-s001.zip › ijms-1670265-supplementary-english.pdf]

International Journal of *Molecular Sciences*

Article

Identifying Novel Osteoarthritis-Associated Genes in Human Cartilage Using a Systematic Meta-Analysis and a Multi-Source Integrated Network

Emily Shorter [1,*], Roberto Avelar [1], Margarita Zachariou [2], George M. Spyrou [2], Priyanka Raina [1], Aibek Smagul [1], Yalda Ashraf Kharaz [1], Mandy Peffers [1], Kasia Goljanek-Whysall [1,3], João Pedro de Magalhães [1] and Blandine Poulet [1]

[1] Department of Musculoskeletal and Ageing Science, Institute of Life Course and Medical Sciences, University of Liverpool, Liverpool L7 8TX, UK; r.avelar@liverpool.ac.uk (R.A.); priyankaraina10@gmail.com (P.R.); aibek@liverpool.ac.uk (A.S.); yalda@liverpool.ac.uk (Y.A.K.); peffs@liverpool.ac.uk (M.P.); kwhysall@liverpool.ac.uk (K.G.-W.); aging@liverpool.ac.uk (J.P.d.M); bpoulet@liverpool.ac.uk (B.P.)

[2] Bioinformatics Department, The Cyprus Institute of Neurology & Genetics, Nicosia 23462, Cyprus; margaritaz@cing.ac.cy (M.Z.); georges@cing.ac.cy (G.M.S.)

[3] Department of Physiology, School of Medicine, The Regenerative Medicine Institute (REMEDI), NUI Galway, H91 TK33 Galway, Ireland

* Correspondence: e.shorter@liverpool.ac.uk

Citation: Shorter, E.; Avelar, R.; Zachariou, M.; Spyrou, G.M.; Raina, P.; Smagul, A.; Ashraf Kharaz, Y.; Peffers, M.; Goljanek-Whysall, K.; de Magalhães, J.P. Identifying Novel Osteoarthritis-Associated Genes in Human Cartilage Using a Systematic Meta-Analysis and a Multi-Source Integrated Network. *Int. J. Mol. Sci.* **2022**, *23*, 4395. https://doi.org/10.3390/ijms23084395

Academic Editor: Gabriela Loots

Received: 22 March 2022

Accepted: 14 April 2022

Published: 15 April 2022

Publisher's Note: MDPI stays neutral with regard to jurisdictional claims in published maps and institutional affiliations.

Copyright: © 2022 by the authors. Submitted for possible open access publication under the terms and conditions of the Creative Commons Attribution (CC BY) license (https://creativecommons.org/licenses/by/4.0/).

Text S1: Supplementary Information on

Multi-Source Information Network Construction and Gene Rankings

Mapping: A background list was compiled with the Ensemble IDs for the protein-coding genes from the human chondrocyte data. We converted the Ensemble IDs to official gene symbols using the R package org.Hs.eg.db [1] for genome-wide annotation for humans. Missing gene names were identified from www.gtexportal.org and added manually for "ENSG00000205583" = "STAG3L1", "ENSG00000221870" = "TMEM257". The OA co-expression network's edgelist was also mapped from Ensemble IDs to their official gene.

Gene List Construction: We formulated three gene lists with two columns corresponding to the gene ID and the respective scores as follows:

1. SEED List: Ranked list of the seed genes identified from the OA meta-analysis, ranked based on *p*-value (of the initial list before filtering). Each gene i received the gene score GS_i per list, calculated based on $GS_{SE} = \frac{L-(R_i-1)}{L}$, where L is the length of the list and R_i is the rank of each gene.
2. SNPs List: Ranked list of genes based on protein-coding SNP variants associated with OA. Each gene received a score GS_{SN} that was calculated based on the expression $GS_{SN} = \frac{\sum_i c_i}{Max_{SN}}$, where c_i represents the confidence level of the prediction for each microRNA targeting the gene (for knee-related variants, $c_i = 1.0$ and for OA-related variants, $c_i = 0.5$) and Max_{SN} represents the maximum value of c_i across all genes.
3. miRs List: Ranked list of the top gene targets of the OA-related microRNAs, based on the number of microRNAs targeting in each gene. We selected here the miRNA experimentally validated gene targets (from miRTarBase) of "miR-140-5p", "miR-150-5p", and "miR-424-3p". Each gene received a score GS_M calculated based on the expression $GS_M = \frac{M_i}{Max_M}$, where M_i represents the total number of microRNAs targeting the gene i and $Max_M = 3.0$ represents the theoretical maximum value.

Network Construction: Three networks were created and analysed using the igraph [2] R package:

1. OA network: the weighted mutual-rank human chondrocyte protein-coding network, filtered for the top 15 most co-expressed genes for each seed.

2. SEED network: We constructed different types of networks for the genes in the seed list from the GeneMANIA tool [3] in Cytoscape [4] with the following options (Species: Homo sapiens, Networks: co-expression, co-localisation, genetic interactions, physical interactions, network weighting: automatic, no additional genes or attributes). The different GENEMANIA networks were merged into one by summing the individual weights for each pairwise gene–gene edge.

3. miRs network: number of common microRNA targeting each pair of genes. We constructed a network based on the knowledge we gathered for the targeted genes by the three key microRNAs identified to be experimentally verified for OA. Each gene-to-gene pair was assigned a weight based on the number of common microRNAs targeting both genes.

Filtering: The background gene list was used as the reference gene list towards which all other sources were compared and filtered. Based on this comparison, two genes were excluded from the SNPs list (DPEP1, BTNL2) and two were excluded from the SEED list (CEACAM4, SRCIN1).

Multi-source Network Integration and Gene Prioritization: Genes were ranked with respect to their importance and involvement in OA based on the collected multi-source data with the Multi-source Information Gain (MIG) characteristic score as previously described [5]. The MIG score is calculated using two scores:

$$MIG = w * MIG_n + (1 - w) * MIG_e \quad (S1)$$

where MIG_nrepresents the normalized integrated n^{th} gene-specific information (i.e. node characteristics) and MIG_e represents the normalised integrated gene–gene information (based on the topology of the multi-integrated super network) and corresponds to the weighted degree of the multi-integrated super network.

For the first term MIG_n, the gene-specific information was extracted from our dataset with:

$$MIG_n = \sum_i w_i^n GS_i,\ i \in \{M, SN, SE\} \text{ where } \sum_i w_i^n = 1 \quad (S2)$$

Here, GS_M is a vector corresponding to the scored gene list from the miRs list, GS_{SN} is a vector corresponding to the scored gene list from the SNPs list, and GS_{SE} is a vector corresponding to the ranked genes from the SEED list. GS_x is in canonical form as it takes values $GS_x \in (0,1]$ for all gene lists.

For the second term MIG_e, an integrated super network was created by implementing a weighted sum of the edge vectors of three gene–gene networks to obtain the composite edge vector of the network. The edge-specific information for each gene G_i was given by the weighted degree (strength) of the integrated network $MIG_e = stregth(G_i)$, where the edges in the integrated network were given by the expression:

$$M_{edge} = \sum_i w_i^e GE_i,\ i \in \{M, OA, SE\} \text{ where } \sum_i w_i^e = 1 \quad (S3)$$

Here, GE_i vectors correspond to edges in each of the three individual networks, i.e., (i) number of common miRNAs targeting each gene pair (M), (ii) the chondrocyte-expression network (OA), and (iii) the meta-analysis GENEMANIA-derived seed gene network, comprised of co-expression, co-localisation, genetic interactions, and physical interactions between seed genes. The edges of the three networks were normalised to their canonical form (0,1] divided by the maximum edge weight for each respective network. All emerging networks were non-directed weighted networks, with edge weights in the 0 to 1 range.

We considered equal contribution to the score of the gene-specific information and of the topology of the integrated gene–gene super network ($w = 0.5$ in Equation (S1)). The weights w_x^n and w_x^e for the respective sources were selected to represent the level of empirical confidence to each source, i.e., $w_M^n = 0.,1$, $w_{SN}^n = 0.4, w_{SE}^n = 0.5$ in Equation (S2) and $w_M^e = 0.1$, $w_{SE}^e = 0.4, w_{OA}^e = 0.5$ in Equation (S3).

References

1. Carlson, M. org.Hs.eg.db: Genome Wide Annotation for Human. R Package Version 3.8.2. 2019. Available online: https://bioconductor.org/packages/org.Hs.eg.db/ (accessed on 13 April 2022).
2. Csardi, G.; Nepusz, T. The igraph software package for complex network research. *Int. J. Complex Syst.* **2006**, *1695*, 1–9.
3. Warde-Farley, D.; Donaldson, S.L.; Comes, O.; Zuberi, K.; Badrawi, R.; Chao, P.; Franz, M.; Grouios, C.; Kazi, F.; Lopes, C.T.; et al. The GeneMANIA prediction server: biological network integration for gene prioritization and predicting gene function. *Nucleic Acids Res.* **2010**, *38*, W214–W220. https://doi.org/10.1093/nar/gkq537.
4. Shannon, P.; Markiel, A.; Ozier, O.; Baliga, N.S.; Wang, J.T.; Ramage, D.; Amin, N.; Schwikowski, B.; Ideker, T. Cytoscape: A Software Environment for Integrated Models of Biomolecular Interaction Networks. *Genome Res.* **2003**, *13*, 2498–2504. https://doi.org/10.1101/gr.1239303.
5. Zachariou, M.; Minadakis, G.; Oulas, A.; Afxenti, S.; Spyrou, G.M. Integrating multi-source information on a single network to detect disease-related clusters of molecular mechanisms. *J. Proteom.* **2018**, *188*, 15–29. https://doi.org/10.1016/j.jprot.2018.03.009.
